# A Real-Time Distance Measurement System for a Digital Twin Using Mixed Reality Goggles

**DOI:** 10.3390/s21237870

**Published:** 2021-11-26

**Authors:** Krzysztof Lalik, Stanisław Flaga

**Affiliations:** Faculty of Mechanical Engineering and Robotics, AGH University of Science and Technology, Al. Mickiewicza 30, 30-059 Kraków, Poland; stanislaw.flaga@agh.edu.pl

**Keywords:** digital twin, intelligent systems, mixed reality, Industry 4.0

## Abstract

This paper presents a new system architecture for controlling industrial devices using Mixed Reality (MR) applications and a new method based upon them for measuring the distance between real and virtual points. The research has been carried out using a physical robot and its Digital Twin (DT). The possibility of controlling them using gestures recognized by Mixed Reality goggles has been presented. The extension of the robot’s environment with a 3D model capable of following its movements and positions was also analyzed. The system was supervised by an industrial Programmable Logic Controller (PLC) serving as an end point for the data sent by the goggles and controlling the movements of the real robot by activating the corresponding control. The results of the preliminary measurements presented here concerned the responsiveness of the system and showing the influence of system parameters in the accuracy of distance estimation between measured points.

## 1. Introduction

The expanding industry presents not only new challenges but also new perspectives on technology. In the era of Industry 4.0, there remains a wide variety of new devices and methods for communicating and connecting them in new ways at our disposal [1,2]. Digitalization is allowing for the development of new manufacturing technologies [3], smart systems [4] and sensory systems [5,6]. In the case of the sensory field, there was also a significant increase in the contribution of intelligence in data processing [7] and in measurement methods [8,9,10].

One of the extremely interesting systems being used with the Industry 4.0 standard is Mixed Reality. This technology makes it possible to extend, e.g., an industrial space full of machines with customized applications showing their status, visualizing occurring processes, and even allowing to control them [11,12]. The use of this technology allows for more flexible process management and support for the operator of a given device. In this case, the operator is equipped with special interactive goggles which can display holograms and information for the user and be the basis for collecting information from the environment around the user [13,14] and transmitting information to higher-level systems [15,16]. This technology is potentially a great improvement and enhancement for process operator support.

Another core pillar of the Industry 4.0 standard is Digital Twin (DT) technology. According to [17], the Digital Twin is a cyber-physical intelligent system for the dynamic presentation of a physical object that integrates the management of distributed information from multiple sensory systems and augmented and mixed reality technology to supervise the operation of the test object and to share and refresh discrete data between real and virtual systems. The combination of Digital Twin and Mixed Reality goggles technologies, along with their advanced sensory circuits, could therefore lead to a new class of intelligent Machine–Human (M2H) interfaces that would enhance the operation and safety of the devices and their collaborating users.

**Figure 1:**
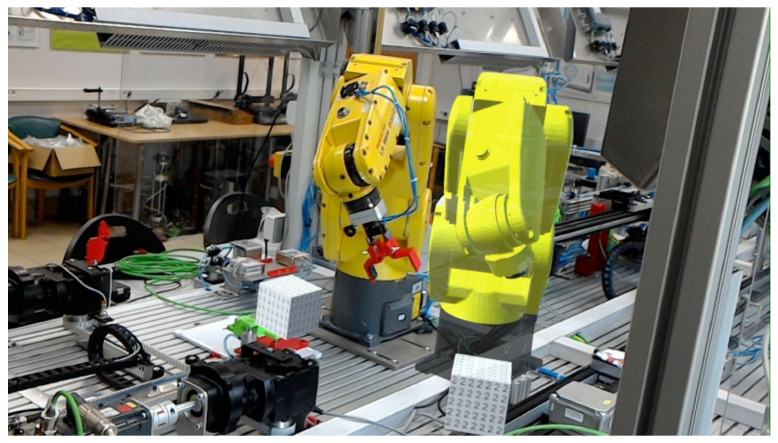
A physical 6-axis robot with its interactive Digital Twin displayed with MR goggles.

This paper describes the architecture and length measurement method for the created interactive Digital Twin for a six-axis robot (Figure 1). The DT can be freely moved, rotated and scaled using gestures by an operator with MR goggles on his head. The entire system architecture has been designed to simultaneously perform control of both the physical robot and its DT. The Digital Twin follows the preset motion of the physical robot.

There are many well-known and effective distance measurement methods based on camera images. From the marker-free methods, it is important to highlight the stereovision measurements [18,19]. In the proposed systems, images from several cameras are compared, and the distance of the obstacle from the scene is calculated from them. The depth is then calculated using disparity maps. The principle of stereoscopic measurement is to place two cameras in parallel and at a known distance from each other and from that compute the distance to the obstacle or marker [20]. For single-camera measurements, however, markers are used [21]. The distance measurement is performed according to the effect of changing the marker size as the distance from the marker increases while keeping the imaging parameters constant. There are also a lot of methods for distance measurement in the infrared spectrum [22]. The measurement principle is based on extracting depth information using two IR channels and an IR transmitter to compute a map of the disparity between the IR channels and transforming this information into a depth map. One of the interesting solutions is presented in [23]. The infrared depth camera extracts the spatial locations of the joints of the observed subjects, and the proposed algorithm finds three points in the floor plane to obtain its equation. This produces an algorithm for recognizing whether a person has fallen on the IR image. The advanced 3D laser scanners [24] cannot be missed in 3D imaging. This is the most powerful solution and offers depths of up to 50 micrometers, but it is also a very expensive solution. As described in this paper, Hololens goggles contain both stereo and infrared cameras. All of the described methods can therefore also be used in them.

This paper presents the concept and preliminary research results for a system to measure the distance between a Digital Twin and real objects using Mixed Reality (MR) goggles. The use of such a system is critical to speeding up design work in the development of modern machinery and equipment. With the real environment at the designer’s disposal, an interactive hologram can be layered to mimic the real device. Such a Digital Twin can be integrated with the existing architecture of control devices, creating, in analogy to the Hardware-in-the-loop technique, a new DT-in-the-loop technique. Such a system will allow, on the one hand, the immediate integration of the real device and, on the other hand, will allow performing tests and measurements with virtual mapping. The software side allows preparing a ready-made solution, e.g., in a PLC, which will control the DT. This software will be ready to control the real device without the need to change the controller. The test side, which is a subject of this article, will allow to safely measure the online distance between DT elements and unknown real environment. This measurement will be possible through the use of Mixed Reality goggles.

## 2. Theoretical Foundation and Related Work

In the current state of technology, three main realities must be distinguished: Virtual Reality (VR), Augmented Reality (AR) and Mixed Reality (MR), while VR technology is not controversial, the division between AR and MR technologies is a contentious issue. VR means total immersion of the user in the virtual world. By donning the appropriate instrumentation, the user can interact with the virtual environment and thus create and receive information using essentially the same mechanisms as a computer. AR technology, on the other hand, allows the user to become less immersed in the virtual world [25]. In this case, AR technology superimposes virtual elements on top of the real environment and thus expands the user’s understanding of the environment and can additionally be used to display additional information. This is where the main axis of the struggle between the nomenclature and division of AR and MR lies. The manufacturer of Hololens goggles, Microsoft^®^, claims that Mixed Reality is a reality in which virtual elements are superimposed on the real world, unlike in AR technology. In AR technology, an image of reality is taken from cameras and then displayed on a device (mobile phone, monitor, TV or non-transparent goggles). Virtual elements are then superimposed on the virtual reality image. In Hololens goggles, transparent displays are present that superimpose holograms on the real world view and in this way, the manufacturer defines a clear distinction between AR and MR. In pure opposition to this distinction are works by [26,27], which claim that no such distinction exists at all. There are also works like [28] which claims MR is a combination of VR and AR. Regardless of the ever-present discussion on Realities and all the controversies, this work takes after the manufacturer that the used goggles belong to the MR group.

Mixed and augmented reality are already widely used in technology. The primary application of each of the mentioned technologies is educational. Excellent methods for the implementation of future practices of full virtual education suitable for the new generation of digitally-oriented student community are described in [29,30]. In these papers, research results and proposals for the application of various VR and AR techniques in training, user support and virtual learning are presented. In the [29,30] excellent methods for implementing future practices of full virtual education suitable for the new generation of digitally-oriented student community are described. In these articles, research results and proposals for the application of various VR and AR techniques in training, user support and virtual learning are presented.

An interesting solution in room mapping is presented in [31,32]. The authors of these papers present the possibility of obtaining a digital representation of large indoor spaces in buildings using Hololens goggles. A geometric evaluation of the data for the obtained mesh in terms of precision and space coverage is presented in detail. However, the described solutions refer only to obtaining a Point Cloud of the existing reality, which classifies them in the area of passive applications, in contrast to the platform proposed in this paper. A similar solution is presented in [33]. In this case, the creation of a digital twin using external RGB-D sensors is presented. In this case, Deep Learning networks were used to analyze the image obtained from these sensors and create an image of the operator skeleton for the first RGB-D sensor and scan the environment of the robot environment. The network-based on this information, decided to slow down or to safely stop the robot. The MR goggles were used in this case to display danger information to the user. Thus, they operated in a semi-active mode as they were used to present the task to be performed and safety information to the user. The information was actively sent from a higher-level system, but the system architecture was not suited for direct interaction with the control on the MR goggles side.

One interesting approach for analyzing the received image is the one presented by [34]. This paper presents an approach in which the images received by CCTV cameras allow computing the optimal trajectory for a drone searching for a landing site during a storm. Image analysis allows the drone to determine the position of the drone and the position of objects that may be attracted to lightning. A neural network can then generate an optimal landing trajectory that takes into account both time to land safely and avoiding potentially lightning-prone areas.

In most cases, MR goggles are used to visualize a given process [25,35]. In these works, the concepts of cyber-physical systems and their combination with web service-based architectures such as the Internet of Things (IoT) are discussed. These works present AR interfaces as systems that are coupled to the Digital Twin of the device and allow real-time information to be displayed to the user. The data can come from both the actual sensory system and the DT of the process. However, it is important to note that this is again a semi-active system in that it actively displays information but does not directly affect process control.

## 3. System Topology

A Digital Twin is defined as a digital representation of the processes, systems and, most importantly, objects that physically exist. In order to create a digital object, it is necessary to have a description of the real object (to replicate the structure) and a communication system allowing the integration of real elements and their digital replicas.

Communication is essential due to the fact without data exchange, processing and analysis it is not possible to accurately mimic the physical object in real-time. The absence of any information about the object would limit the functionality of such an application to just theoretical analysis. The preparation of the Digital Twin, in addition to the creation of a virtual model, also requires the proper configuration of the real object and preparing it to work with the application.

The key components of the proposed system are three devices: MR goggles, a PLC and a physical robot. Communication will take place between them, and they will perform the role of interface, control and execution.

**Figure 2:**
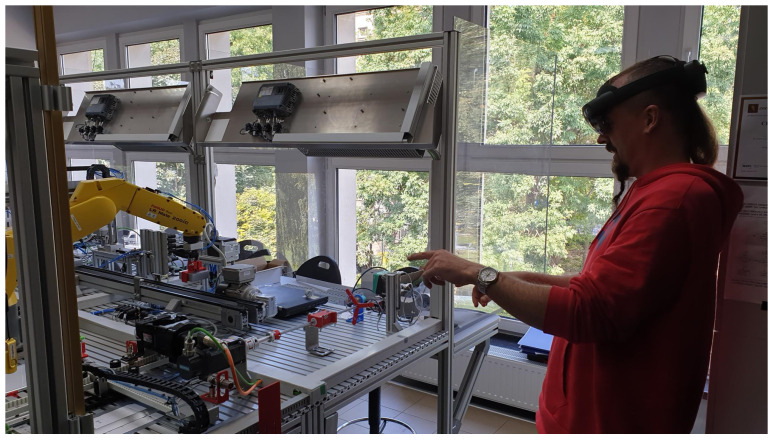
Microsoft Hololens MR goggles on the operator’s head.

As a Mixed Reality device, the first-generation Microsoft HoloLens goggles were used (Figure 2). It is a device that connects the real world with the virtual world by allowing 3D and 2D applications to be overlaid on the physical environment. The control is done using head movements and, above all, gestures, which are tracked and interpreted by cameras. The platform is equipped with a quad-core and quad-threaded Intel Atom x5-Z8100 processor, 2 GB RAM and a wide range of different types of sensors. These include an inertia sensor (accelerometer, gyroscope and magnetometer), four cameras that track head movements, an additional depth camera, two cameras designed purely to track eye movements and a Holographic Processing Unit (HPU). The HPU’s job is to determine its location and orientation relative to the real environment and to build a real-time map of the environment. Data are provided to the user using an integrated display with a 30 degree (horizontal) by 17.5 degree (vertical) field of view.

The unit responsible for controlling the real process and the Digital Twin is a Siemens PLC, model ET 200SP. The robot which is the object of this research is the LR Mate 200iC from Fanuc. It has six rotary joints, making it classified as an anthropomorphic robot. It achieves a repeatability of 0.02 mm with a load of 5 kg, where its own weight is 27 kg. The maximum range is 704 mm, and the mounting options are standard, suspended or angled.

**Figure 3:**
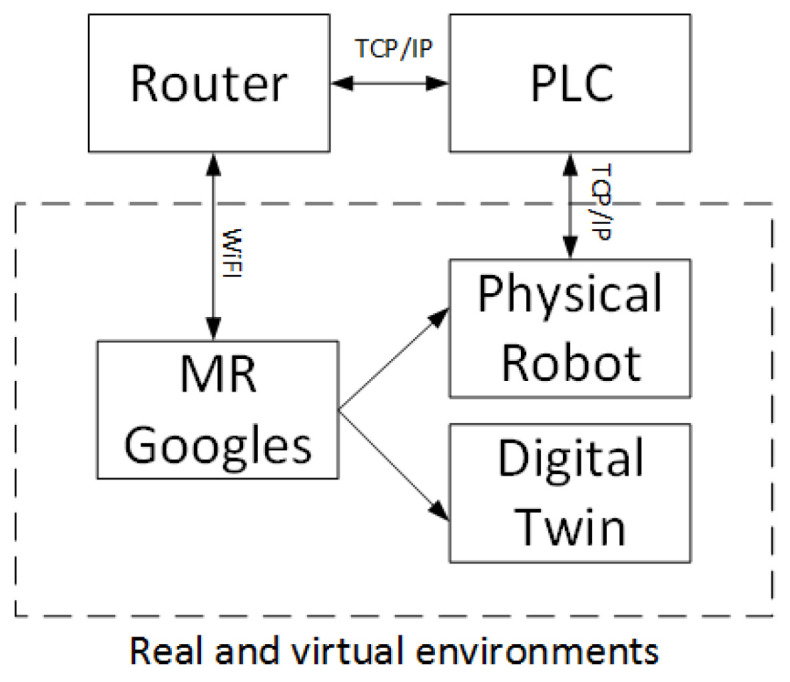
Schematic diagram of the system.

The system communicates according to the diagram in Figure 3. The controller represents the main role here - similar to the server role. On the one hand, it communicates with the MR goggles by receiving information and values of variables processed by them. One of this information is the global position of the effector for DT, which value is sent cyclically to the PLC. The second task is to realize the control of the robot, and through the implemented communication mechanism, it receives information continuously about the angle of rotation of each of the six joints of the physical robot. Knowing the dimensions of the robot and the location of its base, it is possible to determine the global position of the physical robot effector. The controller also has the task to acquire this data.

The controller is also responsible for performing safety tasks. However, DT as a virtual entity is not able to damage the equipment or the user, but it is different from a physical robot. One of the proposed possibilities, which is possible due to the proposed system architecture, is to move the robot by moving its DT in space. Thus, two-way interaction is possible. Either the physical robot forces the DT to move, or the DT forces the physical robot to move. In the second case, a collision is possible. Hence the PLC has the task to prevent this. This is possible in several ways. First are all physical devices—light curtains, external e-stop buttons and integrated e-stops in the teach pendant of the robot. The PLC stops the physical robot immediately when any of these circuits are interrupted. It has also implemented the ability to interrupt the robot using a button on the HMI screen, which will allow the function responsible for stopping the robot from being activated and deactivated during the program run. In this way, the operator is able to observe and archive process parameters and data. The last option is to stop the physical robot with MR goggles. The operator makes a gesture to the PLC to stop the robot immediately.

**Figure 4:**
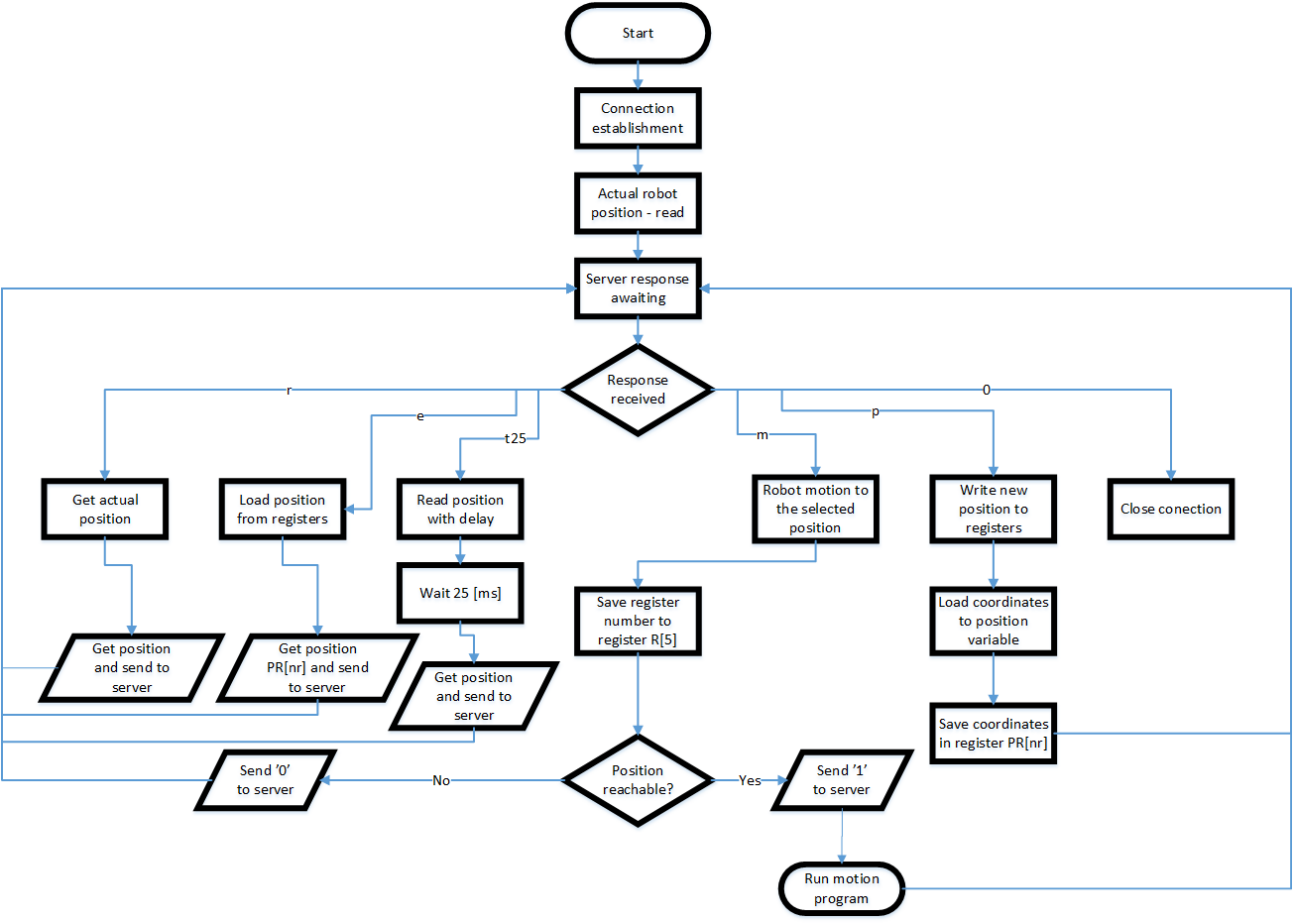
Server–client program activity diagram.

Communication between the MR goggles and the PLC is wireless. The goggles connect to a WI-FI router, which connects to the PLC using TCP/IP protocol. The controller uses the same protocol to communicate with the robot. The implementation of the Ethernet interface, in this case in a client–server architecture, assumes two-way communication. The server is implemented in the PLC. The server listens, waiting for a connection request from the client. A client program imported into a FANUC Robot written in Karel will try to connect to a defined address using TCP/IP when called. There will be two-way communication between the two programs when the connection is established. To enable the movement, it was necessary to write an additional program in the Teach Pendant language. Since the Karel language does not allow control of the robot’s movement, it will under certain conditions call a program to move to a specific position along a path in linear or joint interpolation. The refresh rate of the current position sent to the computer is also an important aspect of the interface. The target frequency was specified to be 10Hz or higher. The server and all clients should be assigned to the same network so that all devices are visible to each other. The tasks of the client program are shown in Figure 4.

**Figure 5:**
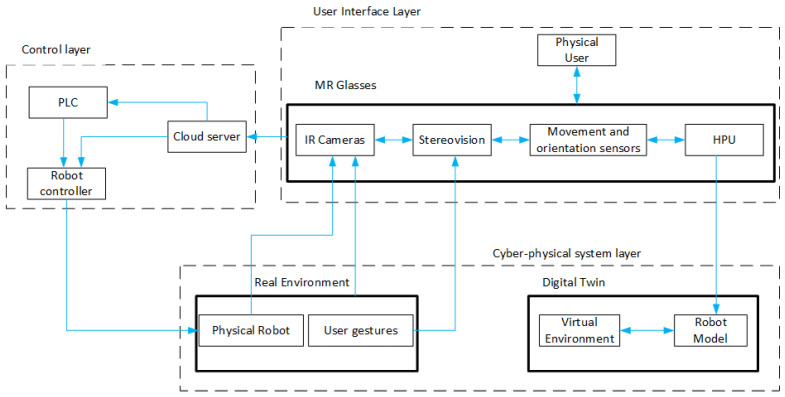
Architecture of a fully active system control and distance measurement interface.

The system architecture (Figure 5) is based on a set of sensors and processors that are integrated into the Hololens goggles. IR cameras are primarily used to measure the depth map of real objects. Additionally, stereo cameras can be used for reference mapping of the real environment. However, in this case, due to the goal of achieving the lowest possible processing time for the MR goggles, these cameras were only used to recognize the user’s gestures and hand position. The measurement of the DT position of the robot and other virtual elements in real space was realized by the HPU. All sensory systems continuously updated the created maps using data from the operator position and orientation measurement systems integrated into the MR goggles. As much raw data as possible were sent to a server in the Cloud so as to reduce processing time. By manipulating with gestures on the virtual representation of the robot model, the information about its spatial configuration has been sent by the server directly to the robot controller or to the PLC managing the robot. In this way, using MR goggles, active interaction with the physical device was possible. The control algorithm was supposed to replicate in real-time on the real robot the spatial configuration of the virtual robot set by the operator with gestures. It should be noted that, due to the real-time paradigm, the presented system architecture aims at the fastest possible data exchange. Therefore, the use of boosters—i.e., point cloud filtering and post-processing algorithms available for Hololens goggles-was abandoned. Possible raw Point Cloud data were provided to the computing system. This allowed for faster data transfer without the additional latency caused by the Edge Computing processing of the Hololens goggles.

## 4. Methodology

The distance measurement mechanism is based on the depth camera. The light emitted by an infrared illuminator has a special structure, which is unique for the position of a pixel, which, knowing the mutual position of the illuminator and the sensor, allows us to unequivocally determine the spatial position of a given point. For this purpose, the same dependencies are applied as in the case of stereovision system—so-called triangulation, i.e., the use of similarity of triangles to find the Z coordinate (depth) of a point. It is also necessary to use an algorithm matching the structures recorded by the sensor to find the disparity value for each point. The depth apparatus of the Hololens device implements amplitude modulated continuous wave (AMCW) time-of-flight (ToF) methods. A beam of modulated infrared light is emitted onto the observed scene. The intermediate transit times of the reflected wave are then recorded. These measurements are processed to generate a depth map. A so-called Depth Map is created, which is a set of Z coordinate values for each pixel in the image. The pixel value in the IR spectrum is proportional to the number of beacons returned from the scene. The camera is equipped with two NIR LEDs to display a wide and near Field of View (FoV). The ToF pixel size is 3.5μm×3.5μm, and the FPS is 24 frames/second.

The accuracy of the instrument is determined by the sum of the systematic and random errors. $E_{sys}$ is a systematic error defined by Equation (1). It is the difference between the measured depth and the actual depth. This error is calculated over multiple frames of a static scene to eliminate depth noise as accurately as possible.

(1) $$E_{sys}=\frac{\sum_{t=1}^{N}d_{t}}{N}-d_{gt}$$

where:

- $d_{t}$ is the measured depth at time moment *t*;
- *N* is the number of frames used for averaging;
- $d_{gt}$ is the actual depth value.

The random error is related to the interference of the scene snapshot. This interference is related to the aging of the array over time. The random error for a static scene $E_{rand}$ is defined as the standard deviation of the depth over time is given by Relation (2).

(2) $$E_{rand}=\sqrt{\frac{\sum_{t=1}^{N}{\left(d_{t}-\bar{d}\right)}^{2}}{N}}$$

where:

- $d_{t}$ is the measured depth at time moment *t*;
- *N* is the number of frames used;
- $\bar{d}$ is the average value calculated over all depth measurements.

**Figure 6:**
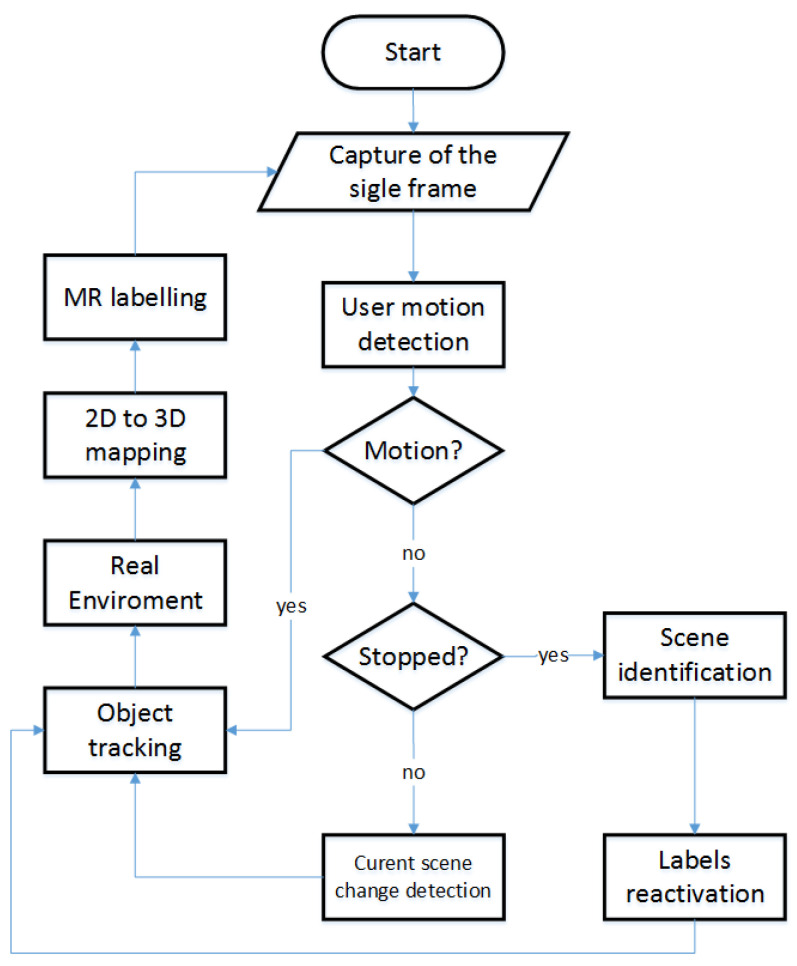
The 5MR system information flow diagram.

The measurement algorithm is shown in Figure 6. Once the MR application is initialized, and a single frame is taken, processing of the resulting image is performed to provide specific frame data. Among others, a grayscale histogram is calculated, and a representation of the observed objects is built. Mapping of 2D detection to 3D space is achieved through a 2D to 3D mapping algorithm. The method involves detecting, using different sets of sensors and cameras, the focal point of the frame and the position and orientation of the camera in world space during the execution of the frame. The focal point position is then converted from scene-related coordinates to world-related coordinates. The intersection of the scene and world meshes at a known camera position results in the ability to determine the global position of a pixel in world space. This procedure is repeated for each pixel in a given frame, thus mapping each frame and assigning a label of 3 coordinates in space for each point. If operator motion is detected, the camera position is updated first, and then only the mapping and intersection of the new real grid with the scene grid from the obtained frame is performed.

**Figure 7:**
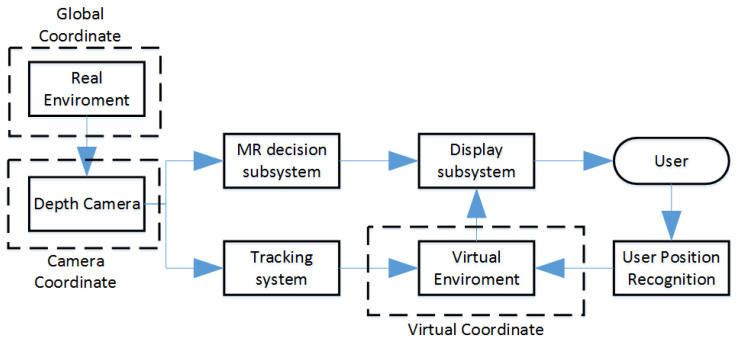
Information flow in MR measurement and display system.

A schematic of the sensory side and the hologram side for MR goggles is shown in Figure 7. The system receives real-time data about the global location and orientation of the camera. Using tracking algorithms, it processes the position information in the digital world for each frame of the real image. The goggles, having the information about the real-world location and global and virtual coordinates, are able to superimpose a 3D hologram of the presented Digital Twin at a specific location. The hologram is then displayed on display and becomes visible to the user.

**Figure 8:**
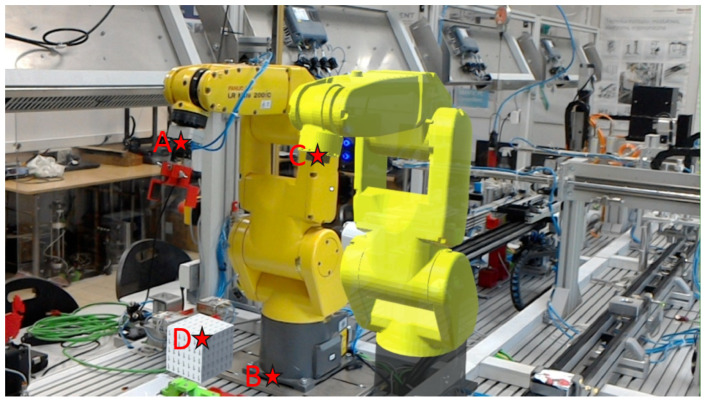
Real view with the digital twin of the robot superimposed, where: A—a point on the actual robot’s wrist; B—a reference point on the actual robot’s base; C—a point on the Digital Twin of the robot; D—a point on the virtual object.

Three types of measurement possible with MR goggles have been proposed. The labeling of the points is shown in Figure 8. The first one is the Real2Real point measurement (points A-B). The second measurement will be on the real point to point at the Digital Twin (points A-C). It will also be possible to measure on the line DT to point on another virtual representation (Points C-D). In order to verify the measurements coming from the digital environment, two alternative distance measurement methods were proposed. For the A-B distance measurement, a Leica DISTO X3 laser distance meter with a range of 150 m and an XYZ coordinate determination accuracy of 1 mm/1 m was used. For reference with virtual scene points, a physical ruler was used. Such a ruler was superimposed on the image observed through the MR goggles and then a photo was taken. The distance between the given points was read from the photo. The reference measurement did not take into account the position of the MR goggles but only the normal projection of a given virtual point on the ruler. These results were then compared with the results obtained from the proposed system. Both the robot and its Digital Twin and the virtual element were moved so that measurements were not made for only one scene-setting. This was possible due to the MR interface created, which allowed the virtual objects and DT to be moved using operator gestures, with the angle data at each joint sent directly to the real robot control. The tests measured various distances between the claimed points ranging from 0.3 m to 1.5 m.

The measurement of the position and distance between points was not just based on one frame. In order to improve the accuracy of the measurement, the distance measurement algorithm was performed in several variants:

- Variant 1: 1 s film, 24 frames of image, observer stationary;
- Variant 2: film of 3 s, 72 image frames, stationary observer;
- Variant 3: film from 10 s, 240 image frames, stationary observer;
- Variant 4: film from 3 s, 24 image frames, moving observer;
- Variant 5: film from 5 s, 72 image frames, moving observer;
- Variant 6: film of 10 s, 240 image frames, moving observer.

**Figure 9:**
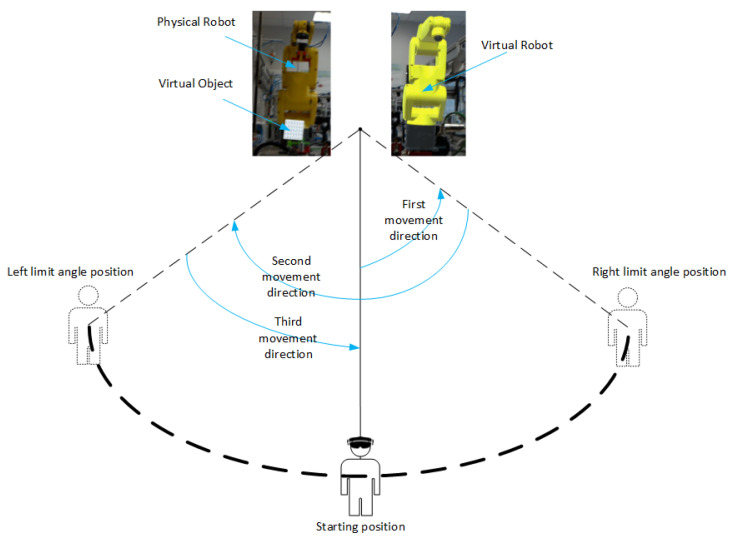
Schematic diagram of an experiment to evaluate the effect of operator motion on the accuracy of distance measurements between test objects.

The movement of the observer was implemented according to the scheme showed in Figure 9. In the beginning, the starting point of the observer was determined as a normal plane to the observed real scene. The observer was not supposed to change the elevation angle when looking at the given object. However, the azimuthal angle of looking at the object was changed. From the starting point, the observer should move to the right in order to deviate the azimuthal angle by $15^{\circ }$, then move to the left to obtain the azimuthal angle of $-15^{\circ }$ and return to the starting position. The final measured distance was the average of the measured distances from each frame of the video. The standard deviation relative to the reference measurement was then calculated.

**Figure 10:**
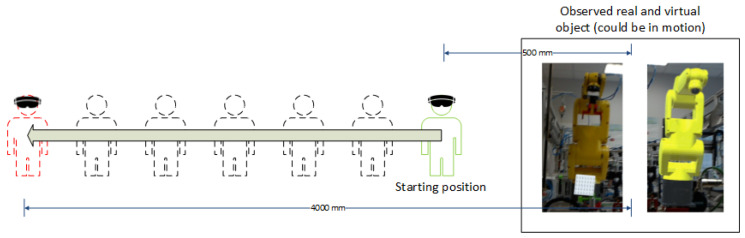
Schematic diagram of an experiment to evaluate the accuracy of distance measurement depending on the operator’s location.

In the version of the measurement shown in Figure 10, the positions of the operator in which the measurement took place are in a straight line towards the scene under study. The operator moves away from the scene under study between 0.5 m and 4 m. At specified distances, the operator tries to be still for a specified period of time, and measurement data is collected. After the data are collected, the operator moves away by another 0.5 m and repeats the measurement procedure.

**Figure 11:**
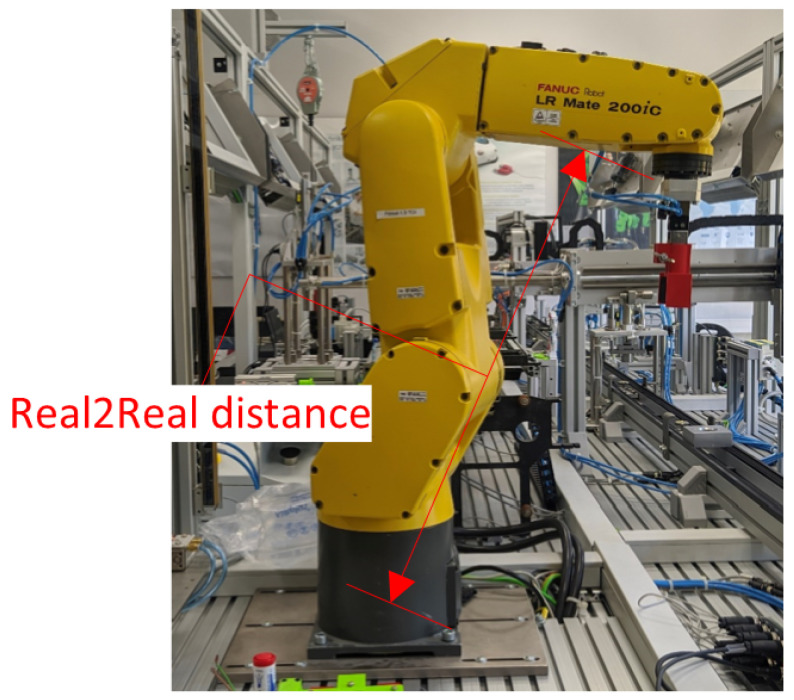
Visualization of the measurement between two real points (Real2Real).

The scheme for measuring the distance between two real points is shown in Figure 11. In this setup, the proposed algorithm did not involve any additional markers. The user declared two points on real objects through gestures, and the system marked and recognized them even when the robot was moving. In this case, active measurement of the distance between the points for the moving robot was not tested, although it was possible. For the tests, in this case, it was more important to determine the effect of the operator’s movement and position in relation to the scene under study. Hence the robot was not moving during the measurements.

**Figure 12:**
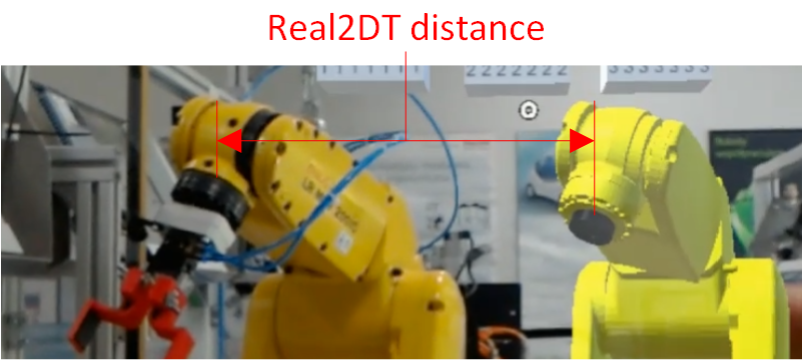
Visualization of the measurement between a real point and the corresponding point on the virtual representation of the robot (Real2DT).

For the distance measurement between the physical robot and its virtual representation (Figure 12), pointing to a point on the real robot automatically resulted in the declaration of the corresponding virtual point on its DT. As shown in Figure 8, both robots were aligned parallel in space with each other. Hence, the value of the distance between these points should remain the same after the movement of the robot. Hence, for the Real2DT measurement, all the presented procedures were collected for 10 different spatial configurations of the robot. The operator performed for the given spatial configuration the procedure shown in Figure 9 and Figure 10. Then using gestures, he repositioned the DT and caused the physical robot to move. Further measurements were performed on each new spatial configuration. The images from all 10 configurations were used to calculate the distance measurement errors without distinguishing by spatial configuration number.

**Figure 13:**
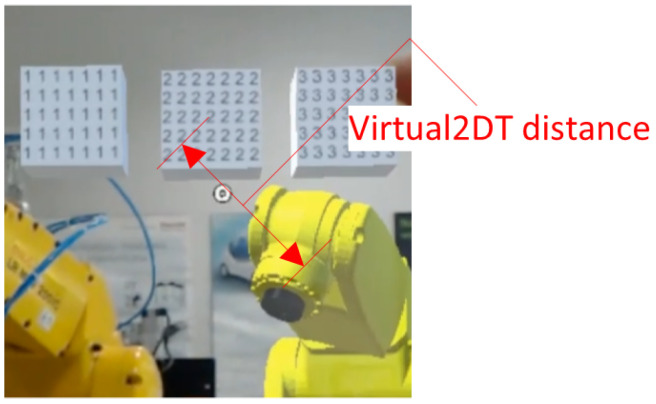
Visualization of the measurement between a virtual point and a point on the virtual representation of the robot (Virtual2DT).

When the distance between virtual elements (Figure 13) was specified, the points between which the distance was measured were declared from within the application and provided to the HPU. The DT of the robot was then overdriven by gestures to a different spatial configuration other than the default. The virtual object was also moved by user gestures to a different position and orientation. The performed procedure allowed to fully confirm that the HPU will not lose the declared points at the objects even when the objects were moved. The displacement of the test objects was followed by the procedures presented in Figure 9 and Figure 10.

## 5. Results

### 5.1. Measurement of System Responsiveness

**Figure 14:**
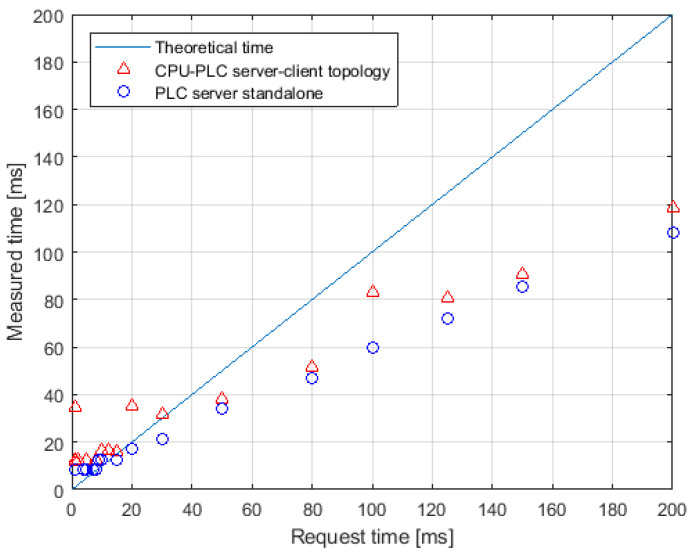
Diagram of the requested and actual response time for the client-external server system and for the system where the PLC is a server.

Figure 14 presents the results of measurements performed on the system simulators. A comparison has been made between the system where an external CPU was used as the server and the PLC as the client and the case where the server was implemented in the PLC itself. It can be seen that in both cases, the connection period of the end device is shorter than the set maximum response time for times above 30 ms. The test shows that the technical assumptions of the refresh rate were met, thus the response period T=50[ms] was assumed, which is equivalent to a frequency f=20[Hz]. In the next step, the response times of the real robot to interaction from DT were checked.

**Figure 15:**
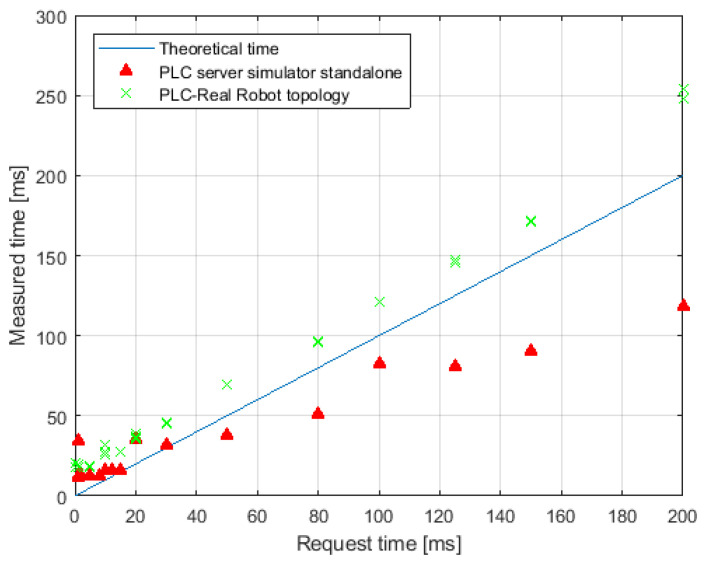
Overview of the actual responsiveness of different system topologies.

The tests of the interface carried out in the physical laboratory allowed to verify its operation in real conditions. The connection was successfully established, and the devices were able to communicate with each other as in the simulated environment. In this case, it was also necessary to verify the measurement of the frequency response in a real configuration. For this purpose, as before, a series of measurements were made using the time function. Each set value was tested on 100 samples. The results are shown in Figure 15. The real-world measurements showed that the physical robot responds in a time greater than that set by the program. Similar to the simulation, a response time of T=50[ms] is achievable, but the query must be programmatically set to a period of 30 ms. The maximum refreshment frequency was obtained by setting the query time to 1 ms and then the real period $T_{real}=18\;\left[\mathrm{ms}\right]$ was achieved, i.e., the frequency $f_{real}=55.5\;\left[\mathrm{Hz}\right]$. The technical assumptions are therefore met also in the real configuration. However, it should be noted that the query from the MR goggles was used as a trigger, and the response of the real robot from this trigger was measured. In reality, the responsiveness of the system depends primarily on the fixed camera rate, i.e., 24 FPS, and the time it takes the algorithm to process the image and the hologram. The HPU processing time is highly dependent on the complexity of both the physical scene and the complexity of the hologram.

### 5.2. Distance Measurement

**Figure 16:**
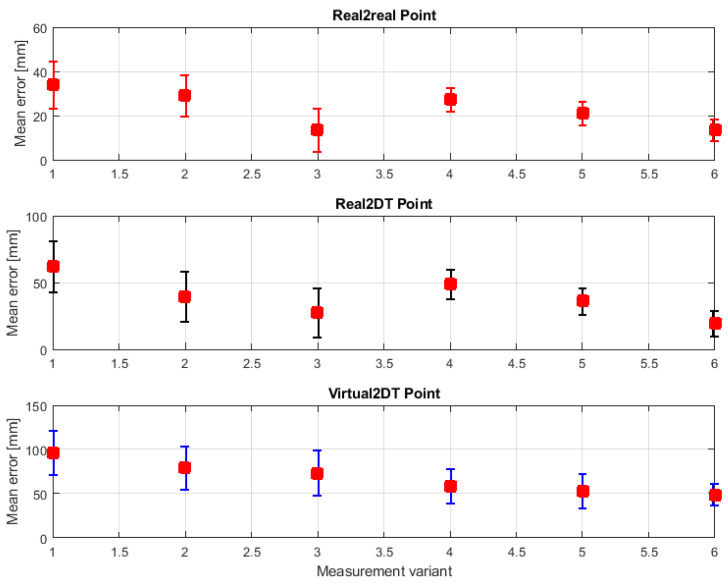
Mean error and its standard deviation for different measurement variants.

The actual part of the study was performed to determine the influence of object type (real/virtual/mixed) on measurement accuracy. The measurement accuracy declared by the manufacturer, which was 10 cm, was also verified. All the measurements performed proved that the actual accuracy is better than the declared one. Figure 16 and Table 1 show the measurement results for six measurement variants. The results clearly show that the lowest distance measurement error for each variant occurs for measuring the distance between two real points and the worst for measuring between two virtual points. The average measurement error for variant 1 is 34.2 mm for Real2Real points and 96.15 mm for two virtual points. These results are easily explained because each virtual object, including the Digital Twin, is calculated by the HPU and then superimposed on reality, resulting in the introduction of additional errors just from the mapping. The introduction of new generations of MR goggles will have the effect of reducing the impact of this error on the results.

Analyzing the data in Table 1, it can be seen that increasing the number of frames from which the mean distance value was calculated caused a significant reduction in the mean error, but also a reduction in the standard deviation of this error for each pair of points. The most interesting effect, however, is the significant improvement in results between variants 1 vs. 4, 2 vs. 5 and 3 vs. 6, so variants with a stationary observer and a moving observer. In each case, the moving observer resulted in improved performance. Furthermore, so, for example, the average error for comparable variants 3 and 6, where to calculate the average used a film of 10 s and 240 frames of the image was for the system of points Real2Real was 13.6 mm with a deviation of 9.89 mm with a stationary observer. For moving observer, it was slightly less, 13.4 mm, but the error spread was drastically reduced and amounted to less than 50% of standard deviation calculated in variant 3. Even better results were observed for distance measurements between virtual objects. There was a reduction in the average error of about 35% and a reduction in the standard deviation of more than 53%. Therefore, the reason for such an effect should be determined. Its first reason is the very architecture of the system described earlier in this paper. The system examines both the position of the MR goggles, the position of the scene, and the position of the holograms. The trigger that triggers the new measurement of the position of these elements is the built-in gyroscopic and accelerometric sensors in the MR goggles. Hence, with the observer stationary, the positions of, for example, the goggles may not be refreshed. Therefore, if the position of the goggles is taken with a certain error, this may affect the accuracy of the calculation of the position of the observed points. The averaging method used will thus add a constant error in the position of the goggles. The movement of the operator, as it were, forces a continuous update of the goggles position and scene measurement, which significantly reduces the mean error and its spread. The second effect, which explains the improvement of dynamic measurement quality relative to static observation, is the effect of the appearance of new edges. The proposed algorithm, when the operator moves, can take into account new edges and points of the scene that were previously obscured by other objects. In this way, the algorithm will be provided with additional grid points that will significantly improve the quality of the obtained results. Thus, the movement of the operator allows for a better knowledge of the 3D scene and, as a result, a better accuracy in determining the position of the operator.

**Figure 17:**
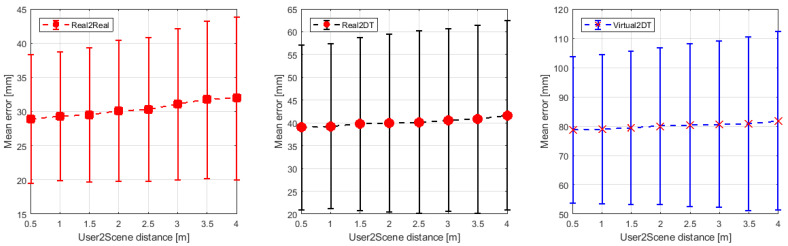
Mean measurement error and its standard deviation for measurements made at different distances from real and virtual objects.

The research experiment also attempted to evaluate the effect of static user distance on measurement error for real/virtual/mixed type objects. The results are presented in Figure 17 and in Table 2. For each object type, an increase in the average length measurement error is noticeable. Obviously, this phenomenon could have been predicted in advance. It is due to the fact that the resolution of the cameras in MR goggles is constant. Hence as the distance from the environment under study increases, there is a larger scene area for the same pixel size. Hence the error in edge and point detection must increase, and there is a degradation in both the mean error and the standard deviation of the mean error. In this case, for the Real2Real point-to-point measurement it increased from 28.91 mm to 31.98 mm at an operator-point distance of 4 m. For the Real2DT measurement it increased from 39.01 mm to 41.66 mm, and for the Virtual2DT measurement it increased from 78.63 mm to 81.84 mm. Obviously, due to the previously discussed mechanisms for creating the Point Cloud in MR goggles, the smallest measurement error occurs for real points and the largest for virtual feature measurements. The standard deviation of the mean error in each case increased by about 20% over a distance of 4 m.

The presented method of measuring the distance between real and virtual elements is prospective. The presented results of preliminary tests indicate the possibility of its implementation in real conditions. However, it is still necessary to look for algorithms for measurement improvement, including Artificial Intelligence algorithms, while waiting for the improvement of hardware technology.

## 6. Conclusions

This paper presents a Mixed Reality interface that allows, on the one hand, for us to control the operation of the Digital Twin and, and on the other hand, to perform online measurements of the distance between real and virtual elements. A method for performing such measurements was presented along with the entire system architecture. The results of the preliminary measurements concerned the responsiveness of the system and the influence of the system parameters on the accuracy of the distance estimation between the measured points. The work enabled to formulate the following conclusions:

- The measurement that has the smallest average error is the measurement of the distance between two real points. Distance measurements to virtual elements are less accurate because the algorithm of superimposing holograms on the real scene introduces an additional layer with HPU, which is a source of further inaccuracies.
- With the interface created, it is possible to control with gestures in a real-time mode both the real systems and the Digital Twin. The introduction of the MR interface also allows for the online integration of those two systems.
- Significant improvements in measurement results have been achieved by bringing the operator into motion. This is a strong added value of the proposed solution because, in different production lines, the operator is always in motion. Hence, he does not have to stop to take measurements, and his movement positively affects the results.

The added value for the presented solution is the creation of a new system architecture that allows the interaction of the real world and the digital twin, as opposed to passive and semi-active systems. In the first case, camera-based devices only allow distance measurement. In the second case, some interaction with the user is additionally possible by displaying to him the information processed by the computer. In the proposed solution, the user’s interaction with the Digital Twin placed in the physical environment allows for direct interaction with the real-world control system. Hence, the created architecture is a fully active user environment capable of both measuring distances (including distances between the physical and virtual worlds) and allowing the realization of the real world control interface by controlling the DT system in mixed reality. Additionally, unlike the well-known applications of Hololens goggles, the proposed distance measurement mechanism allows not only to measure when the user is in motion but also to measure distances when the physical robot or its digital representation is also in motion. The proposed solution is not only for DT visualization, which, according to the available knowledge, is the most common implementation for DT in all Realities (VR, AR, MR). The novelty of the presented system lies in the fact that the digital representation of the device/process can interact with the physical world in real-time mode and also, in a certain sense, simultaneously measure the distance parameters of this interaction.

The implemented interface allows further development of remote control of the robot. The remote control makes it possible to build projects to operate the robot in non-human environments, to perform measurements remotely or to control the robot in mixed reality. Currently, the motion program is limited by setting the upper maximum speed of the robot and the Digital Twin to 20% of the motion speed. It is possible to extend the interface to include speed controls. In order to achieve more complete control of the robot, it will be possible to select the interpolation of the robot motion (joint or linear) by operator gestures. In addition, the interface will be extended with the possibility to determine the workspace with regard to obstacles. Due to this feature, the robot will be able to avoid collision causing movements. The restricted space will be defined by setting up holograms in the virtual space, which will block the robot movement in their space. Therefore, it has become purposeful to define an algorithm measuring the distance between the hologram and the reality, as it will allow already at the design stage to define and verify the work of the robot or its Digital Twin in a way that is safe and does not cause danger to the life and health of the user or damage to the machine or device.

Based on the research, it can be concluded that the presented MR interface is a promising technology. The method still has a significant measurement error, but this will be reduced with new generations of MR goggles. The proposed method should allow to significantly accelerate the process of designing machines and devices and validating their operation at the pre-assembly stage. The Digital Twin technology can thus become one of the pillars of the Industry 4.0 standard.

## Figures and Tables

**Table 1:** Mean error and its standard deviation for different measurement variants.

	Variant 1 Mean Error (mm)	Variant 2 Mean Error (mm)	Variant 3 Mean Error (mm)	Variant 4 Mean Error (mm)	Variant 5 Mean Error (mm)	Variant 6 Mean Error (mm)
A-B	34.20 ± 10.61	29.10 ± 9.51	13.60 ± 9.89	27.40 ± 5.31	21.17 ± 5.27	13.40 ± 4.87
A-C	62.17 ± 19.33	39.44 ± 18.66	27.51 ± 18.65	48.91 ± 11.22	36.04 ± 9.84	19.28 ± 9.46
C-D	96.15 ± 25.96	78.90 ± 25.42	72.62 ± 25.89	58.14 ± 19.36	52.40 ± 19.71	47.94 ± 12.08

**Table 2:** Mean measurement error and its standard deviation for measurements taken at different distances from real and virtual objects.

Distance between User and the Object (m)	0.5	1	1.5	2	2.5	3	3.5	4
A-B	28.91 ± 9.43	29.3 ± 9.51	29.5 ± 9.89	30.1 ± 10.33	30.3 ± 10.52	31.05 ± 11.11	31.75 ± 11.49	31.98 ± 11.92
A-C	39.01 ± 18.13	39.27 ± 18.20	39.76 ± 19.06	39.99 ± 19.62	40.15 ± 20.07	40.62 ± 20.15	40.81 ± 20.68	41.66 ± 20.91
C-D	78.63 ± 25.13	78.91 ± 25.51	79.34 ± 26.31	80.01 ± 26.91	80.36 ± 27.89	80.69 ± 28.47	80.81 ± 29.65	81.84 ± 30.54

## Data Availability

Not applicable.

## Abbreviations

The following abbreviations are used in this manuscript:

DT	Digital Twin
MR	Mixed Reality
VR	Virtual Reality
AR	Augmented Reality
IR	Infrared
RGB-D	Red Green Blue Depth image
PLC	Programmable Logic Controller
M2H	Machine to Human
HPU	Holographic Processing Unit
CPU	Central Processing Unit
FoV	Field of View
ToF	Time of Flight
AMCW	Amplitude Modulated Continuous Wave
